# Characteristics and therapeutic resistance mechanisms of the prostate cancer immune microenvironment: a comprehensive analysis from bench to clinic

**DOI:** 10.3389/fphar.2026.1769271

**Published:** 2026-04-07

**Authors:** Jun Li, Xiong Wang, Xiaoshuang Tang

**Affiliations:** 1 Ankang Central Hospital, Ankang, Shaanxi, China; 2 The Ankang Hospital for Maternity and Child Health, Ankang, Shaanxi, China; 3 Department of Urology, The Second Affiliated Hospital of Xi’an Jiaotong University, Xi’an, Shaanxi, China

**Keywords:** androgen receptor, cold tumor, immune microenvironment, immunotherapy, prostate cancer, therapeutic resistance

## Abstract

The immunosuppressive, ‘cold’ tumor microenvironment (TME) of metastatic castration-resistant prostate cancer (mCRPC) is a primary cause of immunotherapy failure. This review delineates the androgen receptor (AR) signalling pathway as the central orchestrator of therapeutic resistance. AR activation directly suppresses CD8^+^ T-cell cytotoxicity, expands immunosuppressive myeloid and regulatory T-cell compartments, and drives spatial heterogeneity in immune infiltration. We highlight that responsive patient subsets can be identified by specific biomarkers—such as biallelic CDK12 inactivation or DNA damage repair deficiencies—which guide the use of immune checkpoint or PARP inhibitors. Emerging agents, including mutation-specific AR degraders and bispecific T-cell engagers, show potential to reprogramme the TME. However, clinical translation remains challenging, limited by discrepancies between preclinical models and human tumour ecology, coupled with dynamic evolution under therapeutic pressure. Overcoming resistance will require an integrated strategy combining high-resolution spatial multi-omics, advanced disease models, and biomarker-driven trials focused on rational combinations that co-target the AR pathway. Prioritizing this mechanistic approach is therefore critical to advance precision therapy for prostate cancer.

## 1 Introduction and research rationale

### 1.1 Epidemiology and current status of immunotherapy for prostate cancer

The progression of advanced prostate cancer to metastatic castration-resistant prostate cancer (mCRPC)–a lethal stage characterized by profound therapeutic resistance–is heavily influenced by an immunosuppressive tumor microenvironment (TME) (Lin et al., 2023a). Although immunotherapy has transformed oncology, its success in mCRPC remains limited, reflecting the tumor’s “cold” phenotype (Lin et al., 2023b). Immune checkpoint inhibitors (ICIs) show modest (∼4–10%) monotherapy response rates, constrained largely by androgen receptor (AR) signaling which suppresses CD8^+^ T-cell function and upregulates PD-L1. Other modalities face similar TME-mediated barriers: therapeutic vaccines (e.g., sipuleucel-T) exhibit constrained efficacy due to low immunogenicity and antigen escape. Similarly, adoptive cell therapies (e.g., CAR-T targeting PSMA/STEAP1) and bispecific T-cell engagers (BiTEs) are hampered by poor T-cell infiltration, exhaustion, and immunosuppressive networks. AR-targeting agents, such as the degrader bavdegalutamide, show clinical benefit. However, resistance emerges through mechanisms such as AR reactivation and neuroendocrine differentiation, which occurs in approximately 17% of mCRPC cases (Wang et al., 2020; Künzli et al., 2022). Overcoming these challenges requires biomarker-driven stratification and rational combination strategies to reprogram the immunosuppressive TME (Zhang M. et al., 2022).

### 1.2 Clinical challenges of the immunologically ‘cold tumour’ phenotype

The profound resistance of mCRPC to immunotherapy is fundamentally linked to two interrelated concepts: an immunologically ‘cold’ tumor microenvironment and active immune escape. We define a ‘cold tumour’ as a state characterized by sparse infiltration of cytotoxic T lymphocytes and a dominant presence of immunosuppressive cellular and molecular networks (Chen F. et al., 2023). This milieu enables immune escape, which is defined as the collective mechanisms by which prostate cancer cells evade detection and elimination by the host immune system, thereby driving therapeutic resistance. The androgen receptor (AR) signalling pathway is a master regulator of both this ‘cold’ state and immune escape processes (Fagman et al., 2015). The “cold tumor” phenotype–characterized by poor T cell infiltration and an immunosuppressive milieu–is a major barrier to immunotherapy success in prostate cancer (Sellars et al., 2022). This is particularly true in the castration-resistant stage. This underscores the need for novel strategies to inflame the TME, such as bispecific T-cell engagers or mutation-specific AR degraders (Lin et al., 2023b; Pala et al., 2022; Hage Chehade et al., 2024). Notably, a significant subset of metastatic mCRPC cases undergo neuroendocrine transformation, serving as a critical resistance mechanism. These observations underscore the necessity of TME-focused research, which is crucial for unraveling resistance pathways and developing effective therapeutic interventions.

### 1.3 The central role of TME research in deciphering resistance

Investigations into the prostate cancer TME are fundamental to deciphering therapeutic resistance, given this complex ecosystem directly mediates carcinogenesis, invasion, and treatment failure (Lin et al., 2023a). The prostate cancer TME fosters resistance through complex cellular and molecular crosstalk. A central mediator is AR signaling, which drives tumor progression while simultaneously suppressing CD8^+^ T-cell function—a key constraint on immune checkpoint blockade that can be reversed by AR inhibition. This interplay between oncogenic signaling and immune function is further exemplified by genetic alterations such as biallelic CDK12 inactivation, which can sensitize tumors to PD-1 inhibitors (Herati et al., 2022; Soerens et al., 2023). Additionally, STEAP1-targeting BiTEs and AR-guided immune cell manipulation represent promising strategies to overcome resistance by remodeling the TME (Pala et al., 2022).

### 1.4 Current immunotherapeutic modalities and their resistance landscape

The limited efficacy of single-agent immunotherapies in prostate cancer stems from a complex landscape of primary and acquired resistance mechanisms. These mechanisms are often rooted in the unique biology of the TME. For ICIs, dominant resistance factors extend beyond PD-L1 expression. These include: (i) AR-mediated immunosuppression, which transcriptionally suppresses CD8^+^ T-cell function and upregulates PD-L1. This creates a dominant inhibitory axis that can be reversed by AR pathway inhibition (Wang et al., 2024; Chen Z. et al., 2023). (ii) PTEN loss and PI3K pathway activation, present in approximately 50% of mCRPC cases. This promotes an immune-excluded phenotype and correlates with poor ICI response (Chen S. et al., 2023). (iii) Low tumor mutational burden (TMB) and consequent neoantigen scarcity, which limits the substrate for T-cell recognition. Therapeutic cancer vaccines face barriers such as inadequate antigen presentation due to defective dendritic cell function or dysregulated MHC machinery, and active suppression by enriched myeloid-derived suppressor cells (MDSCs) and regulatory T cells (Tregs) within the TME, which inhibit vaccine-primed T-cell expansion and effector function (Broomfield et al., 2025). Resistance to adoptive cellular therapies like CAR-T and redirecting agents like BiTEs is multifactorial: T-cell exhaustion prior to or following infusion renders effector cells dysfunctional; antigen escape *via* downregulation of target antigens (e.g., PSMA, STEAP1) allows tumor cells to evade recognition; and a cytokine-driven immunosuppressive milieu (e.g., TGF-β, IL-10) directly impairs cytotoxic activity and persistence of engineered T cells (Deets and Vance, 2021; Broomfield et al., 2025). Furthermore, physical barriers posed by cancer-associated fibroblasts (CAFs) and aberrant tumor vasculature can limit efficient trafficking and infiltration of both endogenous and therapeutic T cells. Importantly, these resistance mechanisms are not mutually exclusive but are interlinked features of the immunosuppressive TME. This intricate network underscores the necessity for rational combination approaches that simultaneously target the immune-inhibitory pathways (e.g., using ARSi to relieve suppression), remodel the cellular composition of the TME (e.g., depleting MDSCs), and enhance intrinsic immunogenicity (e.g., through epigenetic modulators or targeted radioligand therapy), thereby converting immunologically “cold” prostate tumors into “hot” ones amenable to immune attack. These interconnected resistance mechanisms are direct manifestations of the core cellular and molecular features of the prostate cancer TME, which will be detailed in the following section.

### 1.5 Search strategy and selection criteria

To ensure a systematic and reproducible approach, we conducted a structured literature search in the PubMed database for articles published between January 2020 and December 2025. The search employed the following Boolean combination: (“prostate cancer” [MeSH Terms] OR “prostatic neoplasms” [Title/Abstract]) AND (“tumor microenvironment” [MeSH Terms] OR “immune microenvironment” [Title/Abstract] OR “immunotherapy” [MeSH Terms]). Inclusion criteria were: (i) original research articles or reviews; (ii) publication between 2020 and 2025; (iii) journal impact factor ≥5; (iv) English language; (v) direct relevance to the prostate cancer immune microenvironment and therapeutic resistance. Case reports and editorials were excluded. The initial search yielded 105 articles. To ensure comprehensive coverage, we supplemented these results by manually searching the reference lists of the included articles and by tracking the work of key authors in the field. This iterative process led to the inclusion of approximately 157 core publications. The conclusions of this review are based on a critical synthesis and appraisal of this body of literature.

## 2 Core characteristics of the prostate cancer TME

### 2.1 Immune cell infiltration profiles

The prostate cancer TME is characterized by a signature of “deficient effector T cell infiltration coupled with enriched immunosuppressive cells.” This profile is particularly pronounced in mCRPC, directly contributing to its “cold tumor” phenotype and poor prognosis (Pala et al., 2022). Lineage analysis reveals reduced total tumor-infiltrating lymphocytes (TILs) (Liu D. et al., 2024; Guo R. et al., 2025), indicating a globally impaired adaptive immune landscape. Moreover, severe depletion and functional impairment of CD8^+^effector T cells have been documented (Siddiqui et al., 2023; Ibrahim et al., 2025), directly compromising cytotoxic antitumor immunity. This coincides with the concurrent accumulation of regulatory T cells (Tregs) and myeloid-derived suppressor cells (MDSCs). These changes collectively establish an immunosuppressive milieu that impairs immunotherapeutic responses (Li et al., 2025a; Gagliardi et al., 2025).

### 2.2 Cytokine networks and immunosuppressive signals

An immunosuppressive cytokine network, coordinated by AR signaling and tumor-cell autocrine pathways, is a key mediator of immune evasion and therapy resistance. In mCRPC, AR signaling acts as a master regulator of immunosuppression (Gagliardi et al., 2025). Concurrently, tumor cells induce immune tolerance through autocrine signaling: for example, Ly6d^+^prostate tumor cells secrete the epidermal growth factor family cytokine amphiregulin under androgen deprivation, maintaining survival *via* autocrine loops and indirectly reinforcing the immunosuppressive TME. Strategies targeting AR signaling or autocrine pathways hold significant clinical potential for disrupting immune escape.

### 2.3 Aberrant immune checkpoint expression

Aberrant expression of immune checkpoint molecules, such as PD-1, is a hallmark of immune resistance in prostate cancer. This abnormal expression is primarily driven by dysregulated AR signaling, which establishes an overall immunosuppressive TME. Clinical evidence reveals subtype-specific heterogeneity: the biallelic CDK12 inactivation subtype exhibits a unique immune contexture. Therapeutic strategies include AR axis blockade to reverse CD8^+^T cell suppression and enhance ICI activity, while STEAP1-targeting BiTEs offer potential by directly activating T cells to bypass checkpoint abnormalities. Future efforts should prioritize molecular stratification to optimize combination regimens.

### 2.4 Spatial heterogeneity of tumor-immune interactions

**FIGURE 1:**
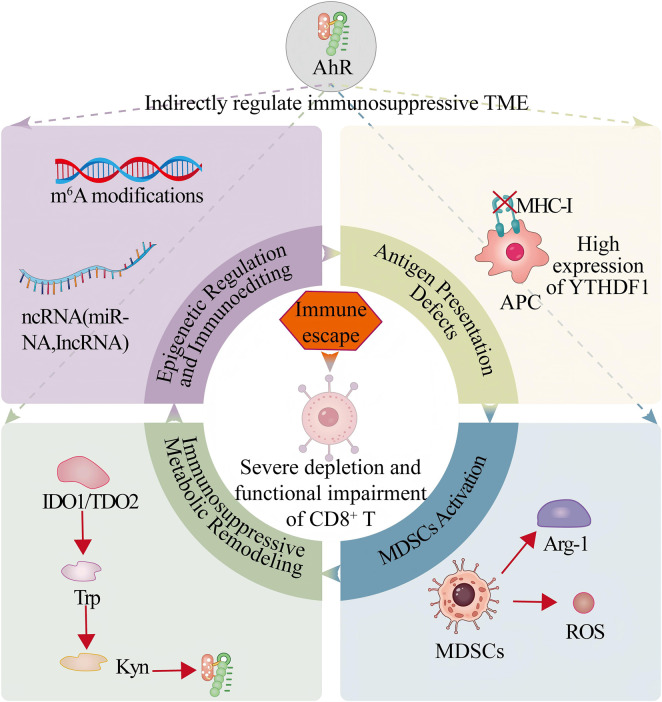
Spatial Heterogeneity of Tumor-Immune Interactions. Legend: Bone metastases enrich M2 macrophages; soft-tissue metastases retain partial CD8^+^ T cell infiltration. AR sustains immunosuppression. Spatial technologies guide stratified therapy.

The spatial heterogeneity of tumor-immune cell crosstalk is a major determinant of variable immunotherapeutic responses in prostate cancer. Characterized by anatomical location dependence, molecular driver specificity, and AR signaling regulation, this heterogeneity can be effectively deciphered using high-resolution spatial transcriptomics (Chen F. et al., 2023). Anatomically, soft tissue metastatic sites of mCRPC exhibit sparse effector T cell infiltration and enriched immunosuppressive cells, which differ significantly from bone metastatic sites and directly impair local immunotherapeutic sensitivity (Chen S. et al., 2023). Molecularly, heterogeneity is closely linked to specific genetic alterations that are discussed in detail in Section 4.1. The AR signaling pathway plays a central regulatory role: its sustained activation suppresses CD8^+^ T cell function, reinforcing local immunosuppression and limiting ICI efficacy. Clinically, this heterogeneity translates to low overall ICI response rates in mCRPC but significant subtype-specific differences. Potential strategies include combining AR axis blockade with ICIs to reverse immunosuppression, while leveraging spatial multi-omics to dissect heterogeneity mechanisms across anatomical sites and molecular subtypes for precise stratified treatment. Future research should focus on the dynamic evolution of spatial heterogeneity to optimize combination strategies (Hirz et al., 2023). The spatial compartmentalization of the immune response is summarized in Figure 1, while a comprehensive comparison of core TME features is provided in Table 1.

**TABLE 1:** Core features of the prostate cancer immune microenvironment.

Category	Key elements	Function/Consequence	Underlying mechanisms	Clinical implications
Immune cell infiltration	CD8^+^ T cells	Antitumor effector function	Negatively regulated by AR signalling; numerically reduced and functionally impaired	Limited response to ICIs; poor prognosis
​	Tregs and MDSCs	Immunosuppression	Enriched; secrete inhibitory factors (e.g., TGF-β, IL-10)	Establish an immunosuppressive TME
Cytokine network	Amphiregulin*etc.*	Autocrine survival signalling	Secreted by tumour cells (e.g., Ly6d^+^) under androgen-deprived conditions	Sustains immune tolerance
Immune checkpoints	PD-1/PD-L1	T-cell exhaustion	Expression indirectly upregulated by AR signalling	Low response rate to ICIs
Spatial heterogeneity	Bone vs. soft-tissue metastases	Distinct immune cell distribution	Driven by anatomical site and molecular features (e.g., CDK12inactivation)	Differential treatment response; necessitates precise stratification
Immune cell infiltration	Total tumor-infiltrating lymphocytes (TILs)	Weakened overall antitumor immune function	Numerically significantly reduced	Insufficient immunotherapy response; poor prognosis

## 3 Key molecular mechanisms of immune escape

Prostate cancer progression is driven by multiple molecular mechanisms of **immune escape**, collectively shaping a highly immunosuppressive TME that impairs antitumor immunity (Figure 1). The defining cellular, molecular, and spatial features that constitute this ‘cold’ phenotype are systematically compared in Table 1, integrating the details discussed in the following subsections.

### 3.1 Antigen presentation defects and T Cell exhaustion

Antigen presentation defects and T cell exhaustion are core mechanisms of immune escape and immunotherapeutic resistance, driven by AR signaling, TME suppression, and key molecular abnormalities. The TME’s “scant effector T cell infiltration + enriched suppressive cells” signature further exacerbates T cell exhaustion *via* networks formed by Tregs, MDSCs, and other immunosuppressive populations. High expression of the RNA m^6^A reader protein YTHDF1 inhibits T cell responses by regulating mRNA stability, while tumor cell autocrine signals such as amphiregulin reshape the TME to aggravate defects (Wang et al., 2024). Molecularly, the potential to reverse T cell exhaustion varies across molecular subtypes, a concept central to patient stratification as detailed in Section 4.1. Therapeutically, AR axis blockade (e.g., androgen deprivation therapy) can enhance ICI activity by improving T cell function, while STEAP1-targeting BiTEs show promise but are constrained by T cell exhaustion. Future research should clarify the specific molecular mechanisms of antigen presentation defects and develop precise interventions combining AR targeting with novel immunotherapies.

### 3.2 Myeloid-derived suppressor cell activation

MDSCs are key drivers of the immunosuppressive prostate cancer TME. They secrete arginase-1 and nitric oxide synthase to deplete arginine—an essential amino acid for T cell activation—and produce reactive oxygen and nitrogen species to directly inhibit T cell proliferation and function. MDSC enrichment correlates strongly with prostate cancer progression and immunotherapeutic resistance. However, while activation pathways such as STAT3 and NF-κB signaling are broadly implicated in cancer-related immunosuppression, their direct mechanistic validation in the context of prostate cancer remains limited, underscoring a key knowledge gap (Sellars et al., 2022), with current understanding inferred primarily from broader immune regulatory networks. MDSC enrichment correlates strongly with tumor progression and immunotherapeutic resistance. The relationship between AR signaling and MDSCs remains an area of active investigation. AR does not appear to directly target MDSCs; however, the observed enhancement of ICI efficacy following AR axis blockade suggests that the AR-driven immunosuppressive TME may indirectly support MDSC function. To date, a direct mechanistic link between AR activity and MDSC biology in prostate cancer has not been experimentally confirmed (Pala et al., 2022). Additionally, AR-targeted therapy resistance-associated transcriptional reprogramming (e.g., ARNTL-mediated neuroendocrine differentiation) may reshape the TME to influence immune cell recruitment and activation, and AR-guided immune cell manipulation strategies imply potential regulatory effects on myeloid cells—though none directly target MDSC activation pathways. The limited efficacy of ICIs in mCRPC is partially attributed to AR-mediated immunosuppression, and MDSCs may synergize with this pathway, but specific activation mechanisms lack direct experimental support (Buck et al., 2023).

### 3.3 Immunosuppressive metabolic remodeling

Tryptophan metabolic reprogramming has been proposed as a significant immune escape mechanism in prostate cancer, though this concept remains primarily supported by preclinical evidence. Tumor cells, MDSCs, and tumor-associated macrophages can express indoleamine 2,3-dioxygenase 1 (IDO1) and tryptophan 2,3-dioxygenase (TDO2), which convert tryptophan (Trp) into the immunosuppressive metabolite kynurenine (Kyn) (Xu et al., 2021; Yang and Wang, 2025; Wang C. et al., 2022; Winquist et al., 2025). Preclinical studies suggest that this process may inhibit T cell proliferation and promote regulatory T cell differentiation *via* aryl hydrocarbon receptor (AhR) activation (Yang and Wang, 2025; Wang B. et al., 2022; Shen et al., 2025), and that IDO/TDO inhibition can partially restore T cell function in model systems (Lopez-Bujanda et al., 2021; Smith et al., 2023; Huttunen et al., 2024). However, clinical translation of strategies targeting tryptophan metabolism has been challenging. Notably, IDO1 inhibitors have failed to demonstrate efficacy in late-stage clinical trials for other cancers. This underscores the risk of extrapolating findings from preclinical models to human disease.

Emerging evidence points to crosstalk between the AR signaling pathway and metabolic processes (Berchuck et al., 2022; Leppänen et al., 2024; Tang et al., 2022). AR signaling, a core clinically validated immunosuppressive driver, may indirectly influence tryptophan metabolism, potentially integrating it into a broader immunosuppressive network. Thus, while tryptophan metabolism contributes to the immunosuppressive landscape, its therapeutic potential likely lies in combination strategies—particularly with AR pathway inhibition—rather than as a monotherapy target. Future research must clarify its role within the AR-dominated ecosystem and identify predictive biomarkers for patient stratification.

### 3.4 Epigenetic regulation and immunoediting

Epigenetic mechanisms are emerging as regulators of immunoediting in prostate cancer, although direct causal evidence remains sparse, and many insights are extrapolated from other malignancies or correlative studies. Reported mechanisms include: the m^6^A reader protein YTHDF1, which may promote immune escape by regulating mRNA stability (Wang et al., 2024); non-coding RNAs implicated in immunosuppressive TME formation (Yang et al., 2023)autophagy involvement in impairing immune-mediated killing (Cui et al., 2023); and histone or DNA modifications that could adapt tumor cells to immune pressure (Yang et al., 2023; Wang et al., 2024). However, the *in vivo* functional relevance and therapeutic vulnerability of these mechanisms in prostate cancer are not yet firmly established.

Notably, epigenetic regulators may interact with core pathways such as AR signaling. AR activity itself can be influenced by epigenetic changes, and conversely, AR can regulate epigenetic modifiers, suggesting a bidirectional relationship. This crosstalk supports the exploration of epigenetic drugs (e.g., HDAC or DNMT inhibitors) in combination with AR-targeted therapies (Pala et al., 2022). Nevertheless, the field must prioritize functional validation in prostate cancer–specific models to distinguish drivers from passenger events and to identify which epigenetic mechanisms offer tractable therapeutic windows. In summary, while epigenetic modulation represents a promising adjunct strategy, its standalone clinical efficacy in prostate cancer immunotherapy requires robust validation, and current evidence justifies its investigation primarily within combination regimens targeting established pathways like AR signaling.

## 4 Clinical translation of therapeutic resistance mechanisms

Resistance to existing therapies—particularly immunotherapy and AR-targeted therapy—is the primary cause of clinical failure in prostate cancer. In-depth understanding of these molecular mechanisms is critical for developing novel therapeutic strategies.

### 4.1 Biomarkers of ICI resistance

**FIGURE 2:**
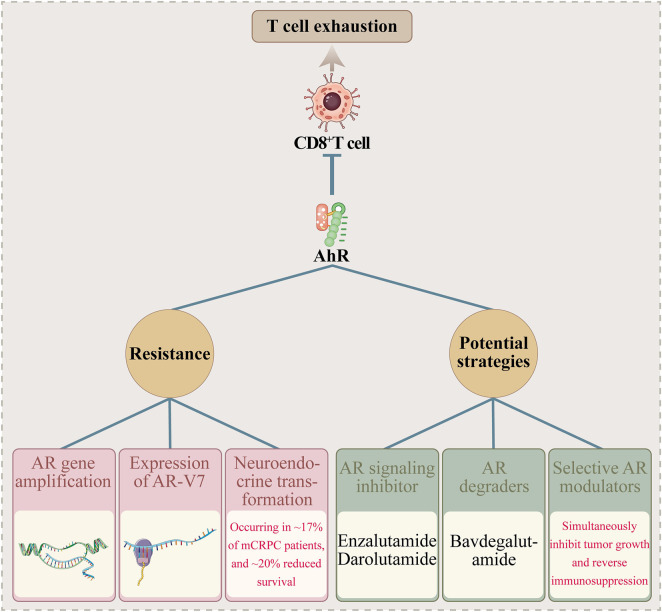
Key Biomarkers of Therapy Resistance in mCRPC. Legend: High AR activity confers ICI resistance, a state reversible by AR signaling blockade. Biallelic CDK12 inactivation and DDR mutations predict sensitivity to PD-1 and PARP inhibitors, respectively. Neuroendocrine differentiation worsens resistance.

ICI efficacy in mCRPC remains modest, as resistance is underpinned by diverse mechanisms and associated with discrete predictive biomarkers. A summary of these key biomarkers, their associated mechanisms, and clinical implications is provided in Table 2. The following biomarkers are of particular clinical relevance:Biallelic CDK12 inactivation: (1) This genetic alteration is associated with pro-immune genetic changes that sensitize tumors to PD-1 inhibitors. In contrast, wild-type CDK12 status is a key biomarker of resistance (Wu et al., 2018). (2), DNA damage repair (DDR) gene mutations (e.g., BRCA1/2): These mutations are established predictors of sensitivity to PARP inhibitors such as olaparib (de Bono et al., 2020). Intriguingly, they may also enhance tumor immunogenicity and thereby potentially modulate ICI efficacy, a hypothesis supported by emerging data that warrants further clinical validation (Yin et al., 2025; de Bono et al., 2020). (3),Treatment-induced neuroendocrine features:Occurring in approximately 17% of mCRPC patients, this transformation is associated with a unique gene expression profile (e.g., ARNTL activation), exacerbates resistance, and reduces survival by nearly 20% (Zhang T. et al., 2022). (4),Other biomarkers:Importin-11 deficiency or PTEN dysfunction (present in ∼50% of mCRPC patients) promotes immune escape *via* PI3K/AKT pathway activation, correlating with low ICI response (Brennen et al., 2021). PD-L1 overexpression on dendritic cells or in enzalutamide-resistant cells can suppress T cell function and is associated with AR pathway inhibitor progression (Winquist et al., 2025; Destefano Shields et al., 2021). These biomarkers reflect the multimechanistic nature of resistance, requiring large-scale clinical validation to optimize patient stratification. Combination strategies, such as AR axis blockade, hold promise for overcoming resistance mediated by these pathways (Destefano Shields et al., 2021). The relationship between key molecular alterations, their impact on therapy, and clinical utility is synthesized in Figure 2, offering an overview of the biomarker landscape that guides precision therapy in mCRPC.

**TABLE 2:** Key biomarkers and mechanisms of therapy resistance.

Biomarker	Associated mechanism	Impact on therapy response	Clinical utility status
High AR signalling activity	Negatively regulates CD8^+^ T cells; upregulates PD-L1	Confers resistance to ICIs; AR blockade can enhance ICI efficacy	Predictive of ICI resistance; combination with AR inhibitors in clinical trials
Biallelic CDK12inactivation	Associated with genomic alterations that may increase immunogenicity	Predicts sensitivity to PD-1 inhibitors	Low frequency, but enables patient stratification
Importin-11Loss	Activates PI3K/AKT pathway; promotes immune evasion	Associated with poor response to ICIs and adverse prognosis	A potential predictive biomarker; requires validation
Therapy-induced neuroendocrine differentiation	Driven by transcriptional reprogramming (e.g., ARNTL activation)	Confers resistance to AR-targeted therapies; shortened survival (∼20% reduction)	Occurs in ∼17% of mCRPC patients; necessitates tailored therapies
DNA damage repair gene mutations (e.g., BRCA1/2)	Increases genomic instability	High response to PARP inhibitors (e.g., olaparib); potential synergy with ICIs	Used for precision therapy; synergy with ICIs under investigation
PTEN dysfunction (∼50% of mCRPC patients)	Activates PI3K/AKT pathway; promotes immune escape	Associated with low ICI response rate; adverse prognosis	A potential predictive biomarker; requires large-scale validation

### 4.2 AR signaling and immunosuppressive crosstalk

**FIGURE 3:**
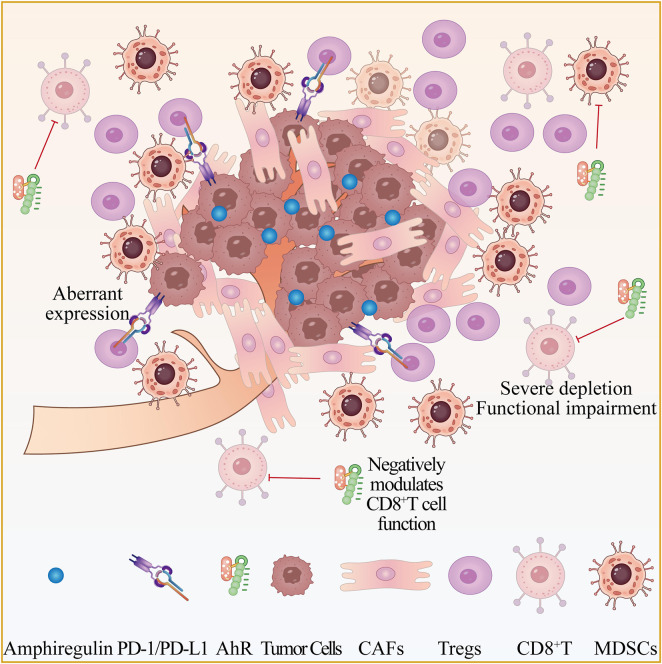
AR-driven immunosuppressive network in prostate cancer TME. Legend: AR suppresses CD8^+^ T cells and enriches Tregs/MDSCs, with PD-1/PD-L1 upregulation inducing T cell exhaustion. This network forms a “cold tumor” with limited ICI response.

As a core clinically actionable driver, AR signaling exhibits critical crosstalk with the immunosuppressive TME, governing both tumor progression and immunosuppressive state formation *via* multiple mechanisms that directly influence treatment responses and resistance (Pala et al., 2022; Chang et al., 2021; Shrestha et al., 2024; Wang Y. et al., 2025; Berchuck et al., 2021). Its core regulatory role is manifested by suppression of CD8^+^T cell-mediated antitumor immunity, inhibiting effector T cell function to promote immune escape—this mechanism is particularly prominent in mCRPC, with androgen axis blockade reversing this suppression to enhance ICI activity (Pala et al., 2022). AR signaling can also directly manipulate host immune cells (e.g., macrophages, T cells) and exert immunoregulatory effects beyond prostate cancer. Furthermore, in addition to its role in immune modulation, AR signaling may inhibit stromal pro-tumorigenic changes in early cancer while indirectly contributing to TME formation. Regarding resistance, AR signaling reactivation is a core mCRPC resistance mechanism, manifested as AR gene amplification, expression of the splice variant AR-V7 (rendering existing antiandrogens ineffective due to lack of a ligand-binding domain), or neuroendocrine transformation (occurring in ∼17% of mCRPC patients, associated with a unique gene profile and ∼20% reduced survival) (Shrestha et al., 2024; Wang J. et al., 2025; Berchuck et al., 2021). Enzalutamide-resistant cells can enhance immunosuppression *via* M2 macrophage polarization to impair ICI efficacy, and glucocorticoid receptor (GR) activation is also linked to AR signaling inhibitor (ARSI) resistance (Ramirez et al., 2023; Serritella et al., 2022; Agostini et al., 2024). Potential strategies include AR degraders (e.g., bavdegalutamide) active in the T878X/H875Y mutation subtype, and the development of selective AR modulators that may simultaneously inhibit tumor growth and reverse immunosuppression (Leppänen et al., 2024). In summary, AR signaling constructs an immunosuppressive TME *via* CD8^+^T cell suppression and immune cell manipulation, with AR targeting combined with immunotherapy representing a key strategy to overcome resistance—requiring optimization based on resistance mechanisms such as neuroendocrine transformation (Agostini et al., 2024). The central role of AR in orchestrating this immunosuppressive milieu, including its suppression of CD8^+^ T cells and enrichment of inhibitory cells, is illustrated in Figure 3, which provides a visual framework for understanding the mechanistic basis of ICI resistance in mCRPC.

### 4.3 Barrier function of cancer-associated fibroblasts

Cancer-associated fibroblasts (CAFs) are core contributors to physical and functional barriers in the prostate cancer TME, promoting therapeutic resistance *via* well-characterized mechanisms (Di Donato et al., 2021; Le et al., 2022; Shadbad et al., 2021; Zheng et al., 2024). Prostate cancer-associated fibroblasts express transcriptionally inactive AR, which can colocalize with HSP90(Heat Shock Protein 90)under androgen stimulation to directly participate in tumor progression *via* non-canonical signaling pathways (Di Donato et al., 2021). More importantly, their barrier effects are dual: on one hand, they secrete immunosuppressive cytokines and chemokines while remodeling the extracellular matrix (ECM), limiting effector T cell infiltration and actively recruiting suppressive immune cells (e.g., MDSCs, Tregs) to reinforce the immunosuppressive TME (Le et al., 2022; Shadbad et al., 2021); on the other hand, they activate signaling pathways such as Wnt/β-catenin to maintain cancer stem cell properties and physically block chemotherapeutic drug and immune cell penetration into the tumor core, ultimately leading to treatment failure (Shadbad et al., 2021). While existing studies confirm AR signaling’s core role in prostate cancer immune regulation—including CD8^+^ T cell suppression and AR axis blockade-mediated ICI enhancement, and potential early stromal regulation to influence TME formation—the direct mechanism of action between AR and CAF barrier function has not been clarified. AR-guided therapy can modulate the TME, but no specific CAF targeting has been identified. Additionally, resistance mechanisms such as limited mCRPC ICI efficacy (Pala et al., 2022), STEAP1-targeting BiTE therapeutic potential, neuroendocrine differentiation (Zhang Y. et al., 2022), and DDR gene mutation-associated PARP inhibitor responses show no direct links to CAF barrier function. In summary, CAFs form a core therapeutic barrier *via* immunosuppression, physical obstruction, and signal regulation, with their interaction with pathways such as AR remaining unclear. Targeting CAFs to break this barrier may represent a key direction to overcome resistance, requiring further in-depth research.

## 5 Novel diagnostic and prognostic evaluation systems

Prostate cancer’s high heterogeneity challenges traditional diagnostic and prognostic methods, driving the development of novel evaluation tools based on TME characteristics.

### 5.1 Clinical value of immune scoring systems

Immune scoring systems significantly improve prostate cancer prognosis prediction and therapeutic stratification accuracy by integrating TME and molecular features, demonstrating key clinical value (Han et al., 2023; Yi et al., 2025; Fenor De La Maza et al., 2022; Ochoa et al., 2025). The Tumor Immune Contexture Score (TICS), which integrates eight immune features (including T cell infiltration density and suppressive cell ratios), effectively optimizes biochemical recurrence-free survival (BCRFS) prediction after radical prostatectomy (Han et al., 2023). The recently proposed Cuprotosis scoring system quantifies cuproptosis-related gene expression, revealing strong associations with TME regulation, therapeutic resistance, and prognosis—high scores correlate with immunosuppressive TME formation and serve as an independent prognostic indicator for clinical stratification (Yi et al., 2025). Additionally, biomarkers such as PD-L1 expression levels, T cell infiltration gene expression profile (TcellinfGEP) scores, and SOX2 have been validated to predict mCRPC patient survival outcomes (Fenor De La Maza et al., 2022; Ochoa et al., 2025). The clinical utility of these scoring systems aligns with the core role of TME and molecular features: the prostate cancer TME (e.g., T cell activity) directly influences disease progression and treatment responses (e.g., AR signaling-mediated CD8^+^T cell suppression, reversible by AR axis blockade) (Pala et al., 2022), and quantitative immune feature assessment enables precise identification of patients likely to benefit from combination therapy. Meanwhile, molecular stratification evidence—such as biallelic CDK12 inactivation predicting PD-1 inhibitor sensitivity and BRCA1/2 mutations guiding PARP inhibitor use—supports the rationale for integrating multi-dimensional features into scoring systems. Although the relevant studies do not focus on specific scores such as the tumor immune microenvironment, the prognostic value of immune and molecular features has been well recognized, and systems like TICS achieve multi-feature integration to provide precise guidance for personalized therapies (e.g., immune targeting, combination therapy). Large-scale clinical validation is required for widespread application.

### 5.2 Multi-omics biomarker panel prediction models

To overcome the limited predictive accuracy of single biomarkers, multi-omics biomarker panel prediction models enable precise assessment of prostate cancer metastasis risk, treatment response, and prognosis by integrating multi-dimensional data—with machine learning algorithms providing key support for model optimization (Li et al., 2025b; Chen S. et al., 2023; Bronte et al., 2023; Lyu et al., 2022; Boufaied et al., 2024; Zhang L. et al., 2025). Leveraging databases such as The Cancer Genome Atlas (TCGA), researchers have constructed immune-centric prognostic models integrating genomic instability, dynamic TME features, and resistance heterogeneity data, significantly improving predictive performance (Li et al., 2025b). A systematic study evaluated 15 algorithms and 30 gene expression features across 10 prostate cancer transcriptomic datasets, identifying an optimal panel model with performance superior to traditional clinical parameters such as Gleason score (Chen F. et al., 2023; Bronte et al., 2023). Additionally, models including hypoxia-immune-related gene risk signatures, cross-omics metabolic biomarkers (e.g., lactate accumulation), and Franken algorithm-mediated immune-stromal cell clustering analysis achieve personalized disease progression prediction by quantifying the spatial distribution and functional status of TME components (e.g., Tregs, MDSCs) (Lyu et al., 2022; Boufaied et al., 2024; Zhang L. et al., 2025). The effectiveness of these models is supported by multi-omics biomarker clinical value: genomically, DDR gene mutations (e.g., BRCA1/2) predict olaparib responses (Ramirez et al., 2023), and biallelic CDK12 inactivation mutations indicate PD-1 inhibitor sensitivity; immunologically, AR signaling suppresses CD8^+^ T cell function (reversible by blockade), providing a theoretical basis for incorporating TME features into models (Pala et al., 2022); additionally, Importin-11 deficiency and neuroendocrine subtype-unique transcriptional profiles expand biomarker sources for models (Li Z. et al., 2025). In summary, machine learning-optimized multi-omics panel models—integrating genomic, immune, metabolic, and other multi-dimensional features—outperform traditional parameters, serving as core tools for personalized prostate cancer stratification. Clinical validation of existing single-omics biomarkers further confirms the rationale of this integrated strategy.

### 5.3 Advances in dynamic TME monitoring technologies

Core advances in dynamic prostate cancer TME monitoring focus on spatial multi-omics analysis and non-invasive monitoring innovation, combined with intelligent imaging and precise molecular quantification—enabling real-time tracking of TME heterogeneity and evolution to support dynamic treatment strategy adjustments (Zhang G. et al., 2025; Shenasa et al., 2025; Guo W. et al., 2025; Goldberg et al., 2021; Li J. et al., 2025). Spatial multi-omics technologies overcome traditional limitations: Digital Spatial Profiling (DSP) quantifies 35 immune biomarkers in tissue microarrays, mapping local immune landscape profiles to provide high-dimensional prognostic data (Shenasa et al., 2025); single-cell mass cytometry (CyTOF(Cytometry by Time-of-Flight)) simultaneously quantifies 36 protein biomarkers, achieving high-precision clustering of 1.6 million cells and clarifying associations between immune/stromal cell subsets and clinical prognosis (Zhang G. et al., 2025). Non-invasive monitoring technologies enable dynamic assessment: CD93 expression-based tumor vascular ultrasound imaging evaluates TME status, and cell-free chromatin immunoprecipitation sequencing (cfChIP-seq) tracks aggressive tumor molecular evolution *via* circulating DNA epigenetic features (Guo W. et al., 2025; Goldberg et al., 2021; Li N. et al., 2022). Extended technologies further enrich monitoring dimensions: AI-assisted MRI improves diagnostic accuracy and efficiency in identifying TME features, and PET/CT facilitates oligometastatic patient stratification and disease burden assessment to guide local interventions (Ng et al., 2025; Murphy et al., 2017). Molecular monitoring strategies are crucial for guiding targeted therapies. These include: sequencing DNA damage repair (DDR) genes (e.g., BRCA1/2) to predict responses to PARP inhibitors–a strategy now integrated into standard practice for selected patients; detecting T878X/H875Y mutations to guide the use of AR degraders like bavdegalutamide, representing a paradigm of mutation-specific therapy; and assessing CDK12 mutation status to identify patients who may benefit from PD-1 inhibitors. Furthermore, assessment of CDK12 mutation status helps identify the small subset of patients who may benefit from PD-1 inhibitors, illustrating the power of genomic stratification (Naz and Petri, 2023). Immune monitoring focuses on AR activity analysis (predicting ICI responses) and assessment of STEAP1-targeting bispecific engager-mediated immune cell interactions (Pala et al., 2022). Current challenges include inadequate intraductal carcinoma detection technologies (Bernardino et al., 2025), and future efforts should integrate multi-omics data and leverage AI to enhance monitoring real-time performance (Dotto et al., 2025). In summary, existing technologies form a multi-dimensional monitoring system encompassing “spatial analysis - non-invasive tracking - molecular validation,” with clinical translation requiring large-scale validation to address heterogeneity challenges.

## 6 Innovative therapeutic strategies

**FIGURE 4:**
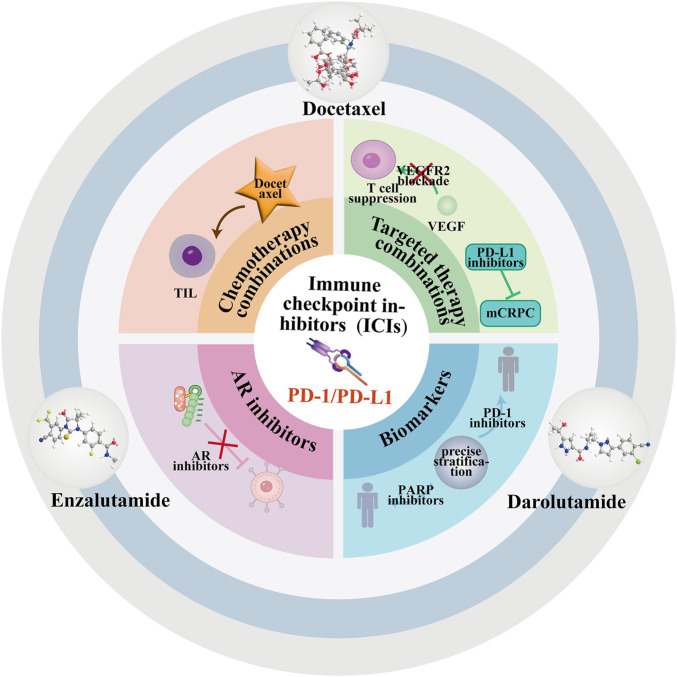
Multi-Dimensional Therapeutic Strategies for Prostate Cancer. Legend: Combination therapy (ICIs + AR antagonists/chemotherapy), metabolic reprogramming, epigenetic modulators, and BiTEs/CAR-T target immunosuppressive TME. Multi-omics enables precise stratification.

Prostate cancer immunotherapy breakthroughs require innovative strategies tailored to its immunosuppressive TME. A multi-pronged approach encompassing these strategies is depicted in Figure 4, which conceptualizes how targeting different layers of the TME can work synergistically. The mechanisms, representative approaches, and current status of these innovative strategies are comparatively outlined in Table 3, serving as a reference that complements the detailed discussion in each subsection. Below, we discuss four key directions: combination therapy, metabolic remodeling, epigenetic regulation, and personalized therapy (Figure 2).

**TABLE 3:** Comparison of novel therapeutic strategies and their mechanistic basis.

Therapeutic strategy	Representative approaches	Mechanism of action	Current status/Challenges
Combination therapy	ICIs + AR antagonists (e.g., darolutamide) or chemotherapy (e.g., docetaxel)	AR blockade reverses immunosuppression; chemotherapy induces immunogenic cell death	Phase III trials show overall survival benefit; optimal timing and sequencing needed
Metabolic reprogramming	TLR5 agonists or ketogenic diet with 1,3-butanediol	Modulates tryptophan metabolism; reduces microenvironment acidity; enhances T-cell function	Preclinical efficacy; combination with ICIs is being explored
Epigenetic modulators	HDAC inhibitors (e.g., panobinostat)	Reverses epigenetic silencing in T cells; enhances antigen presentation	Shows synergy with ICIs in models; application in prostate cancer remains limited
Personalized vaccines and cell therapy	STEAP1-targeted BiTEs or CAR-T; personalized neoantigen vaccines	Directly activates T cells; overcomes low immunogenicity	Early-phase trials show promise; challenges include overcoming immunosuppressive TME
Multi-omics-guided stratification	Integration of genomic and immune features (e.g., TICS score)	Enables precise identification of patient subgroups likely to benefit; allows dynamic monitoring	Relies on AI and spatial technologies; represents a future direction for precision oncology
Personalized vaccines and cell therapy	Engineered exosomes (miR-424 knockdown)	Enhances ICI efficacy	Effective in colorectal cancer models; a potential direction for prostate cancer therapy

### 6.1 Combination therapy: ICI synergy with targeted therapy/chemotherapy

ICIs (e.g., anti-PD-1/PD-L1) show limited monotherapy efficacy in prostate cancer (Pala et al., 2022; Zhang L. et al., 2025; Chen Z. et al., 2023; Sridaran et al., 2023). Synergistic combination strategies of ICI with targeted therapy/chemotherapy provide a key path to overcome this limitation. Chemotherapy combinations: docetaxel-based chemo-hormonal therapy reshapes the TME by increasing TIL numbers and attenuating immunosuppressive signals—significantly enhancing anti-PD-1 efficacy in mouse models, confirming chemotherapy’s ability to induce immunogenic changes for synergy (Ma et al., 2022). Targeted therapy combinations: VEGFR2 blockade inhibits VEGF-mediated T cell suppression, and combined with PD-L1 inhibitors effectively suppresses MYC/p53-driven mCRPC progression (Hawley et al., 2023). AR inhibitors represent core combination partners: the ENZAMET Phase III trial showed enzalutamide combined with standard therapy prolongs metastatic hormone-sensitive prostate cancer (mHSPC) survival, and the ARAMIS Phase III trial confirmed darolutamide delays mCRPC metastasis with excellent safety. Both agents create favorable conditions for ICIs by relieving AR-mediated immunosuppression, requiring only combination timing optimization to avoid antagonism (Kundu et al., 2024). Biomarkers enable precise stratification: biallelic CDK12 inactivation mutation carriers may be sensitive to PD-1 inhibitors, and DDR gene mutation (e.g., BRCA1/2) carriers are sensitive to PARP inhibitors—with theoretical ICI synergy potential (Yu et al., 2023). Resistance challenges require attention: Approximately 17% of mCRPC patients develop treatment-induced neuroendocrine differentiation with poor prognosis, necessitating targeted combination regimens; additionally, STEAP1-targeting BiTEs show promise, expanding combination strategy directions. In summary, combination therapy core is immunosuppression relief *via* chemotherapy and targeted therapy (particularly AR inhibitors), with biomarker stratification and regimen optimization key to improving efficacy.

### 6.2 Metabolic microenvironment remodeling

Metabolic dysregulation—such as lactate accumulation and enhanced tryptophan metabolism—is a preclinically implicated driver of prostate cancer immune escape, with the potential to shape an immunosuppressive TME and promote tumor progression (Li et al., 2024; Li J. et al., 2025). Metabolic pathway targeting interventions can effectively reverse this state and synergistically enhance antitumor immunity. Specifically, Toll-like receptor 5 (TLR5) agonists reshape tumor-associated myeloid cell function *via* toll-like receptor five activation to significantly enhance immune responses (Lin et al., 2023a); periodic ketogenic diets combined with 1,3-butanediol supplementation improve anti-PD-1 efficacy and reduce neutrophil infiltration by lowering TME acidity and regulating epigenetic mechanisms (e.g., histone acetylation) (Zhang L. et al., 2025); PIKfyve-targeted autophagy inhibition (e.g., compound ESK981) converts “cold tumors” to “hot tumors” by increasing cytotoxic T cell infiltration and enhancing ICI efficacy (Qiao et al., 2021). These metabolic interventions may synergize with existing modalities, such as AR antagonists which alleviate T-cell suppression, and biomarker-guided immunotherapies (Hirz et al., 2023; Buck et al., 2023). Treatment-induced resistance challenges require vigilance—such as neuroendocrine differentiation (Approximately 17% of mCRPC patients) (Zhang M. et al., 2022)—with its interaction with metabolic dysregulation remaining unexplored. In summary, metabolic pathway targeting combined with AR blockade, immune targeting, and other strategies can break immune escape *via* multi-dimensional TME remodeling, with molecular stratification providing a key basis for precise combination therapy (Qiao et al., 2021).

### 6.3 Epigenetic modulator application potential

Epigenetic modulators (largely in preclinical development) enhance prostate cancer immunogenicity *via* precise chromatin structure modification, demonstrating significant therapeutic potential. Their core mechanisms revolve around immune-related epigenetic regulation, forming a multi-target intervention system. Histone deacetylase inhibitors (HDACis) such as panobinostat relieve T cell epigenetic silencing to promote interferon-γ secretion, exerting synergistic antitumor effects with ICIs(Zhang G. et al., 2025; Zhang et al., 2024); DNA methylation modulators such as hypomethylating agents (e.g., decitabine) reactivate tumor antigen expression, with preclinical studies confirming improved immunotherapeutic responses (Kiviaho et al., 2024); novel strategies targeting chromatin remodeling complex components (e.g., ARID1A) inhibit inflammation-mediated immune escape pathways to enhance immunotherapeutic sensitivity (Li Y. et al., 2022). Epigenetic modulators hold synergy potential with established therapies, including AR-targeted agents and immunotherapies, potentially converging on a remodeled, more immunogenic TME (Pala et al., 2022). In summary, epigenetic modulators enhance immunogenicity *via* HDAC inhibition, DNA methylation regulation, and chromatin remodeling targeting. Their synergistic application with AR blockade, immune targeting, and other strategies holds promise, providing new therapeutic directions for refractory subtypes such as mCRPC.The key challenge lies in identifying predictive biomarkers for patient stratification and managing the potential toxicity of combination regimens.

### 6.4 Personalized vaccines and cell therapy

Addressing the core challenge of low prostate cancer immunogenicity, personalized immunotherapies have achieved key progress—forming a multi-strategy system centered on vaccines, cell therapy, and engineered vectors—while requiring combination with TME regulation to improve efficacy (Agostini et al., 2024; Zanvit et al., 2023; Zhang M. et al., 2022; Wang C. et al., 2022; Jiang et al., 2024; Armitage et al., 2021). Tumor vaccines: DNA vaccines or dendritic cell vaccines targeting prostate-specific antigens (e.g., PSA, PAP) have demonstrated clinical trial safety, but their antigen presentation efficiency requires immune adjuvant enhancement—with breast cancer research suggesting potential GM-CSF inhibitor applications (Agostini et al., 2024; Zanvit et al., 2023; Zhang T. et al., 2022). CAR-T cell therapy: targeting prostate cancer-specific antigens (e.g., PSMA, STEAP1) shows clear antitumor activity in locally advanced models—the core challenge is overcoming the immunosuppressive TME, and combination with MDSC-targeted drugs (e.g., Indoleamine 2,3-Dioxygenase (IDO) inhibitors) significantly improves T cell persistence (Wang B. et al., 2022; Jiang et al., 2024). Engineered exosome strategies: modified tumor exosomes with miR-424 knockdown enhance ICI efficacy in colorectal cancer models, providing a new direction for prostate cancer therapy (Armitage et al., 2021). The inherent low immunogenicity of prostate cancer, driven by mechanisms such as AR-mediated immunosuppression, necessitates that personalized immunotherapies be combined with TME-modulating strategies. This includes co-targeting immunosuppressive pathways (e.g., AR signaling) and employing biomarker-guided patient selection. In summary, personalized therapies overcome low immunogenicity *via* specific antigen targeting, with large-scale clinical trials required to validate efficacy. Combination with AR blockade, molecular stratification, and other strategies will further enhance their clinical translational value (Table 3).

## 7 Hierarchical evaluation of immunosuppressive mechanisms: distinguishing clinical Reality from preclinical promise

A critical barrier in prostate cancer immunotherapy is the conflation of two types of evidence: mechanisms supported by robust clinical data, and those that remain primarily preclinical observations or speculative hypotheses. Disentangling this hierarchy is essential for prioritizing therapeutic targets and designing rational clinical trials. To provide a clear and actionable framework, we stratify the evidence for major immunosuppressive mechanisms in Table 4, categorizing them as clinically validated, translational, or primarily preclinical. The following sections discuss this hierarchy in detail.

**TABLE 4:** Evidence hierarchy for key mechanisms in prostate cancer immune evasion and therapeutic resistance.

Mechanism/pathway	Evidence level	Key supporting findings and current status
Androgen receptor (AR) signaling as master immunosuppressive regulator	Level 1: Clinically validated	AR activity suppresses CD8^+^ T-cell function; AR-axis blockade (e.g., enzalutamide, darolutamide) improves ICI efficacy in clinical trials; AR-driven signatures correlate with poor immunotherapy response
Biallelic CDK12 inactivation	Level 1: Clinically validated	Genomic alteration associated with increased tumor immunogenicity and sensitivity to PD-1 inhibitors in defined clinical cohorts
DNA damage repair (DDR) mutations (e.g., *BRCA1/2*)	Level 1: Clinically validated	Predict sensitivity to PARP inhibitors (e.g., olaparib); potential synergy with ICIs under investigation in clinical trials
Therapy-induced neuroendocrine differentiation	Level 2: Translational/Mechanistically defined	Occurs in ∼17% of mCRPC; driven by transcriptional reprogramming (e.g., ARNTL); confers resistance to AR-targeted therapies and correlates with poor survival. Robust clinical histopathological correlation
Myeloid-derived suppressor cell (MDSC) infiltration and function	Level 3: Preclinical support	Functional immunosuppression (arginase-1, ROS) well-demonstrated in models; enrichment correlates with progression/resistance in patient samples. Direct mechanistic links to AR signaling not yet conclusively proven in prostate cancer
Tryptophan metabolism (Ido1/tdo2-kynurenine-ahr axis)	Level 3: Preclinical support	Pathway inhibition reverses immunosuppression in preclinical models; however, clinical trials of Ido1 inhibitors in other cancers failed, suggesting contextual dependency. Direct prostate-cancer-specific clinical evidence is limited
Epigenetic regulation (e.g., YTHDF1, ncRNAs, autophagy)	Level 3: Preclinical support	*In vitro* and *in vivo* studies support roles in immune escape and therapy resistance; promising synergy with ICIs in models. Clinical translation remains at early stages
Cancer-associated fibroblast (CAF) barrier function	Level 3: Preclinical support	CAFs promote immune exclusion, ECM remodeling, and suppressive cytokine secretion in models. Interaction with core pathways like AR signaling remains unclear
Metabolic remodeling (e.g., lactate, hypoxia)	Level 3: Preclinical support	Metabolic modulation (e.g., PIKfyve inhibition, ketogenic diet) enhances immunotherapy in models. Clinical evidence in prostate cancer is emerging but not yet definitive
STEAP1-targeting BiTEs	Level 2: Translational/Mechanistically defined	Strong preclinical efficacy; early-phase clinical trials show promise. Represents a rationally designed immune-redirection strategy

Evidence Hierarchy: Level 1 (Clinically Validated): prospective trials or validation in large cohorts; Level 2 (Translational): strong clinicopathological correlation and preclinical mechanistic insight; Level 3 (Preclinical Support): compelling experimental models awaiting definitive clinical confirmation; Level 4 (Hypothetical): theoretical proposals (not assigned to major mechanisms in this review).

### 7.1 The clinically validated core: AR signaling as the master regulator

The androgen receptor pathway stands as the central, clinically validated immunosuppressive mechanism in prostate cancer. Its role in suppressing CD8^+^ T-cell function is well-documented, and critically, AR-axis blockade (e.g., with enzalutamide or darolutamide) has been shown to enhance the activity of immune checkpoint inhibitors in clinical trials. This establishes a direct causal link from mechanistic understanding to clinical intervention. Similarly, genetic alterations like biallelic CDK12 inactivation have been validated in clinical cohorts as predictors of sensitivity to PD-1 inhibitors, defining a clear biomarker-defined subgroup (Fagman et al., 2015).

### 7.2 Translational and mechanistically defined pathways

Mechanisms such as therapy-induced neuroendocrine differentiation occupy a strong translational tier. They are defined by clear histopathological correlation in patient samples, a well-characterized transcriptional driver (e.g., ARNTL), and a direct association with clinical resistance and poor survival. Novel agent classes like STEAP1-targeting Bispecific T-cell Engagers (BiTEs) also reside here, with compelling preclinical efficacy and emerging early-phase clinical data supporting their mechanistically rational design.

### 7.3 The preclinical arena: promising but unverified hypotheses

A significant portion of the discussed landscape, including tryptophan metabolism reprogramming, epigenetic immunoediting, MDSC activation pathways, CAF-mediated barrier functions, and metabolic microenvironment remodeling, is primarily supported by preclinical evidence. While *in vitro* and *in vivo* studies are often compelling and suggest synergistic potential with existing therapies, their direct causal role and therapeutic vulnerability in human prostate cancer remain inadequately proven. The failure of IDO1 inhibitors in late-stage clinical trials for other cancers serves as a cautionary tale about the risks of extrapolating from model systems without robust human validation.

### 7.4 Synthesis and forward path

We posit that the AR signaling pathway functions as a non-redundant, master regulator of immune evasion, with many other putative mechanisms likely acting as contingent or secondary effectors within an AR-dominated ecosystem. This hierarchical model has immediate clinical implications: effective immunotherapy will almost certainly require co-targeting the AR axis. Future research must rigorously test whether preclinical mechanisms operate independently or are downstream of AR signaling through correlative clinical studies (e.g., linking metabolite levels to AR activity) and functional co-targeting experiments. By clearly stratifying evidence, we can transform a fragmented list of observations into a coherent, actionable paradigm for overcoming therapeutic resistance.

## 8 Current challenges and future outlook

### 8.1 Preclinical-human TME discrepancies

A major translational barrier is the failure of preclinical models to fully capture the complexity of the human prostate TME. This discrepancy directly contributes to the limited clinical efficacy of immunotherapies that show promise in these models (O'Melia et al., 2022; Zhu et al., 2023; Dong et al., 2025; Li et al., 2023). Traditional murine models poorly recapitulate key features of the human prostate TME, including its spatial heterogeneity, “cold” phenotype, and critical molecular mechanisms such as AR-driven immunosuppression. low T cell infiltration, limited antigen presentation efficiency, and an immunosuppressive TME enriched with high levels of immunosuppressive factors. While patient-derived xenografts (PDXs) and humanized mouse models can partially approximate human biological characteristics (O'Melia et al., 2022; Zhu et al., 2023; Dong et al., 2025; Li et al., 2023). they still fail to fully recapitulate core ‘cold tumor’ features of the human TME, the direct consequences of this discrepancy remain significant: CAR-T cell therapy, ICIs, STEAP1-targeting BiTEs, and other preclinically promising therapies face bottlenecks in prostate cancer due to inadequate TME recapitulation, resulting in limited clinical efficacy (Li X. et al., 2022). To bridge the translation gap, future efforts should prioritize humanized model or organoid system development to approximate human biological characteristics (Xiang et al., 2025), while leveraging molecular stratification strategies (e.g., DDR mutation-based PARP inhibitor beneficiary selection and combining TME regulatory approaches (e.g., AR blockade to enhance ICI activity) to optimize therapies (Pala et al., 2022). Novel strategies (e.g., BiTEs) require validation in more complex simulation systems to improve clinical translation efficiency.

### 8.2 The need for precise immunotherapeutic response prediction

Prostate cancer patients exhibit significantly lower ICI response rates compared to those with solid tumors such as melanoma and lung cancer, highlighting the urgency of developing precise predictive tools (Li et al., 2023; Agarwala et al., 2024; Kane et al., 2025). Tumor immune microenvironment (tumor immune microenvironment (TIME)) heterogeneity is a core efficacy-influencing factor, and the lack of standardized assessment systems currently limits predictive accuracy (Sipe et al., 2022; Luo et al., 2024). Existing potential predictive factors have clear limitations: genomic alterations (e.g., biallelic CDK12 inactivation, microsatellite instability) can enhance ICI sensitivity but have extremely low prevalence in prostate cancer (San-Jose Manso et al., 2024), their extremely low prevalence in the general prostate cancer population severely restricts their utility as broad-scale biomarkers. AR signaling reduces ICI efficacy *via* CD8^+^T cell suppression, with AR expression or mutation status holding predictive potential (AR blockade enhances ICI activity) (Pala et al., 2022; Dotto et al., 2025); Importin-11 deficiency is associated with abnormal PTEN localization and poor prognosis, potentially indirectly affecting immune escape—though its predictive value requires validation. Multi-omics integration analysis has emerged as a breakthrough direction: prediction models such as TICS—constructed by integrating transcriptomic and spatial transcriptomic data, and incorporating parameters such as immune cell spatial distribution, MDSC abundance, and stromal cell activity—exhibit superior performance (Luo et al., 2024; Zhou et al., 2024; Andersen et al., 2021). Future efforts should address standardized assessment bottlenecks, leveraging AI-assisted MRI analysis combined with circulating tumor DNA (ctDNA) dynamic monitoring to integrate imaging features and molecular evolution information for pre-treatment response stratification and on-treatment resistance early warning (Zhang et al., 2024; Dotto et al., 2025; Zhang and Liu, 2025); simultaneously, expand sample sizes to validate the predictive value of low-incidence biomarkers (e.g., CDK12 mutations), and incorporate key factors (e.g., AR status) into multi-omics models—combining synergistic strategies (e.g., AR blockade) to optimize stratification precision.

### 8.3 Overcoming tumor heterogeneity: technical pathways

Prostate cancer’s high spatiotemporal heterogeneity is a core therapeutic resistance mechanism, with significant differences between primary and metastatic lesions, and within individual lesions—bone metastases highly express M2-like macrophage signature genes and are enriched in immunosuppressive cytokine networks, while soft tissue metastases retain partial T cell infiltration capacity (Fan et al., 2025; Yu et al., 2024; Conway et al., 2022). Single-cell sequencing further reveals intralesional heterogeneity in tumor-immune/stromal cell interactions, leading to local immunosuppressive TME formation (e.g., T cell exhaustion zones) (Zhou et al., 2024; He et al., 2021; Zhang and Liu, 2025). Addressing this challenge requires the development of precise analytical and targeted intervention technologies: high-resolution spatial transcriptomics (e.g., GeoMx DSP) can localize immunosuppressive hotspots (Zhou et al., 2024; Ramel et al., 2021); microfluidic tumor chips can simulate physicochemical gradients (e.g., hypoxia, acidosis) to decipher dynamic TME evolution (Xiang et al., 2025; Li et al., 2025a); targeting cancer stem cell niches (e.g., regulating PUM1/CLOCK transcription factors) can weaken immune escape capacity (Dai and Liu, 2021; Dianat-Moghadam et al., 2023). Simultaneously, molecular stratification and signal regulation need to be linked: AR signaling suppresses CD8^+^T cell function, with AR blockers (e.g., darolutamide, enzalutamide) improving immune responses—while ARNTL-mediated neuroendocrine transformation exacerbates resistance (Zhang T. et al., 2022); biallelic CDK12 inactivation and DDR gene mutations (e.g., BRCA1/2) guide precise PD-1 inhibitor and PARP inhibitor (olaparib) application, respectively (Fan et al., 2025); novel therapies (e.g., STEAP1-targeting BiTEs, T878X/H875Y mutation-targeted AR degraders such as bavdegalutamide) also address heterogeneous subtypes. In summary, integrating high-resolution analytical technologies with molecular stratification interventions is key to overcoming heterogeneity-mediated resistance.

### 8.4 Cross-cancer immunotherapy translation

Despite prostate cancer’s uniqueness—such as AR signaling-mediated immunosuppression (Kane et al., 2025; Zhu et al., 2023; Peng et al., 2022)—translating immunotherapeutic strategies from other cancers remains valuable. Common targeting directions have shown potential: TLR5 agonists reverse tryptophan metabolism-mediated immunosuppression in pancreatic cancer, and this strategy may reshape the prostate cancer metabolic TME (Hao et al., 2021; Duhan and Smyth, 2021); kidney cancer vascular normalization therapy has confirmed that anti-angiogenic drugs (e.g., anti-VEGFR2 antibodies) improve T cell infiltration, with their combination with ICIs entering prostate cancer clinical trials (Lamplugh and Fan, 2021; Hu et al., 2022); EZH2 inhibitors enhance antigen presentation in lymphoma, and high EZH2 expression in prostate cancer is associated with a cold immune phenotype, suggesting application potential (Dai and Liu, 2021; Peng et al., 2022). Cross-cancer strategies require prostate cancer-specific optimization: personalized neoantigen vaccines developed based on other cancers need adaptation to its low mutation burden (Wang Y. et al., 2025; Zanvit et al., 2023); oncolytic viruses (e.g., OH2) need to overcome the prostate stromal diffusion barrier (Xu et al., 2025; Li J. et al., 2025). Additionally, T cell-directed therapy universal mechanisms (e.g., BiTEs such as STEAP1-targeting) and molecular stratification experience (e.g., biallelic CDK12 inactivation, DDR gene mutations) provide translation support. Future core directions include establishing cross-cancer TME databases to identify conserved resistance pathways (e.g., hypoxia-HIF1α axis) (Hao et al., 2021; Cheng et al., 2025; Ghosh et al., 2022), while deepening AR signaling cross-cancer conservation research to promote rapid translation of common targeting drugs. Combining with prostate cancer-specific features (e.g., AR blockade, neuroendocrine transformation resistance) to optimize combination strategies will achieve precise translation.

### 8.5 Bridging the preclinical–clinical gap: biomarker-driven trial design

The repeated failure of mechanistically promising therapies in late-stage clinical trials highlights a critical translational gap in prostate cancer immunotherapy. This disconnect often stems from the inability of preclinical models to fully replicate the complex, AR-driven immunosuppressive tumor microenvironment (TME) and the spatially heterogeneous “cold” phenotype characteristic of human metastatic castration-resistant prostate cancer (mCRPC) (Sellars et al., 2022). This problem is compounded because early-phase trials frequently enrol molecularly unselected patient populations. This dilutes efficacy signals in the subgroups that might actually benefit (Sellars et al., 2022; Mulè et al., 2024). To transform this paradigm, future clinical development must adopt biomarker-driven trial designs that integrate multidimensional stratification. This includes: (i) genomic markers such as biallelic CDK12 inactivation (predictive of PD-1 inhibitor sensitivity) and DNA damage repair (DDR) gene mutations (e.g., *BRCA1/2*, guiding PARP inhibitor use), which define subsets with distinct therapeutic vulnerabilities (Chen Z. et al., 2023; Liu G. et al., 2024); (ii) immune and molecular profiling using tools like the Tumor Immune Contexture Score (TICS) or AR activity signatures to quantify the immunosuppressive landscape and predict synergy with AR-axis blockade; and (iii) spatial multi-omics (e.g., Digital Spatial Profiling) to decipher local resistance niches. Such an integrated framework enables the rational prioritisation of patient subgroups for specific combinations: men with CDK12-inactivated mCRPC are prime candidates for PD-1 inhibitors combined with AR antagonists; those with DDR deficiencies may derive enhanced benefit from PARP inhibitor and immune checkpoint inhibitor combinations; and patients with AR-high, immune-excluded tumours could be targeted with mutation-specific AR degraders (e.g., bavdegalutamide for T878X/H875Y mutations) alongside immunomodulatory agents. Embedding these biomarkers into adaptive, mechanism-focused trial designs will shift the paradigm from empirical treatment to precision enrichment, ultimately accelerating the development of effective combinatorial regimens that co-target dominant resistance pathways such as AR signalling and immune evasion.

## 9 Future research perspectives

Future progress in overcoming prostate cancer therapeutic resistance will depend on a convergent, multi-disciplinary approach that moves beyond descriptive characterization to mechanistic targeting and dynamic clinical translation. Critical priorities include: (1) Deeper dissection of spatial and temporal heterogeneity through integrated single-cell and spatial multi-omics (e.g., spatial transcriptomics, multiplexed imaging), coupled with artificial intelligence-driven modeling, to map immune evasion trajectories and identify tractable niche-specific vulnerabilities. (2) Development of physiologically relevant preclinical models—such as humanized immune system mice, patient-derived organoids with autologous immune components, and microfluidic “tumor-on-chip” systems—that faithfully recapitulate the human prostate TME, including its AR-dominated immunosuppressive network and stromal-immune crosstalk. (3) Refinement of dynamic, non-invasive biomarker platforms (e.g., ctDNA methylation profiling, circulating immune cell phenotyping) to monitor TME evolution in real time, predict early resistance, and guide adaptive therapy. (4) Rational design of combination strategies that co-target AR signaling with immune-modulatory agents (e.g., myeloid-targeted therapies, epigenetic modulators, metabolic inhibitors) in biomarker-selected populations, informed by mechanistic studies of pathway crosstalk. (5) Translational learning from other immunologically “cold” cancers to identify conserved resistance pathways while tailoring approaches to prostate-specific drivers, such as AR variant activity and treatment-induced neuroendocrine differentiation. Ultimately, bridging these frontiers will require close collaboration between basic researchers, computational scientists, and clinical trialists to transform mechanistic insights into personalized therapeutic paradigms.

## 10 Conclusion

Prostate cancer remains a profound clinical challenge, largely due to its immunosuppressive, AR-driven tumor microenvironment that renders it resistant to conventional immunotherapies. This review synthesizes evidence establishing androgen receptor signaling as a central orchestrator of immune evasion—directly suppressing CD8^+^ T-cell function, enriching regulatory and myeloid-derived suppressor cells, and fostering a spatially heterogeneous “cold” niche. While biomarker-guided strategies (e.g., targeting CDK12-inactivated or DDR-deficient subsets) and novel therapeutic modalities (e.g., AR degraders, bispecific engagers) offer promise, translation is hampered by gaps between preclinical models and human TME complexity, along with dynamic tumor evolution under treatment pressure. Future progress will require an integrated strategy that combines high-resolution spatial profiling, human-relevant disease models, and AI-driven biomarker integration to tailor combination therapies across the disease continuum. By prioritizing AR axis modulation within a multidimensional immunotherapeutic framework, future research can transform the management of advanced prostate cancer from empirical to precision medicine, ultimately improving patient outcomes.
